# Methane mitigation potential of phyto-sources from Northeast India and their effect on rumen fermentation characteristics and protozoa *in vitro*

**DOI:** 10.14202/vetworld.2018.809-818

**Published:** 2018-06-19

**Authors:** Luna Baruah, Pradeep Kumar Malik, Atul P. Kolte, Arindam Dhali, Raghavendra Bhatta

**Affiliations:** 1 Energy Metabolism Laboratory, ICAR-National Institute of Animal Nutrition and Physiology, Bengaluru - 560 030, Karnataka, India; 2 Faculty of Biotechnology, Jain University, Bengaluru, Karnataka, India; 3 ICAR-National Institute of Animal Nutrition and Physiology, Bengaluru - 560 030, Karnataka, India

**Keywords:** methane, phyto-sources, protozoa, rumen fermentation, tannin

## Abstract

**Aim:**

The aim of the study was to explore the anti-methanogenic potential of phyto-sources from Northeast region of the country and assess the effect on rumen fermentation characteristics and protozoa for their likely inclusion in animal diet to reduce methane emission.

**Materials and Methods:**

Twenty phyto-sources were collected from Northeast state, Assam, during March to April 2014. Phyto-sources were analyzed for their tannin content followed by screening for methane mitigation potential using *in vitro* system. The effect of tannin on methane production and other fermentation parameters was confirmed by attenuating the effect of tannin with polyethylene glycol (PEG)-6000 addition. About 200 mg dried phyto-source samples were incubated for 24 h *in vitro*, and volume of gas produced was recorded. The gas sample was analyzed on gas chromatograph for the proportion of methane in the sample. The effect of phyto-sources on rumen fermentation characteristics and protozoal population was determined using standard methodologies.

**Results:**

Results from studies demonstrated that *Litchi chinensis, Melastoma malabathricum, Lagerstroemia speciosa, Terminalia chebula*, and *Syzygium cumini* produced comparatively less methane, while *Christella parasitica*, *Leucas linifolia*, *Citrus grandis*, and *Aquilaria malaccensis* produced relatively more methane during *in vitro* incubation. An increase (p<0.05) in gas and methane production from the phyto-sources was observed when incubated with PEG-6000. Entodinimorphs were prominent ciliates irrespective of the phyto-sources, while holotrichs represented only small fraction of protozoa. An increase (p<0.05) in total protozoa, entodinimorphs, and holotrichs was noted when PEG-6000 added to the basal substrate. Our study confirmed variable impact of phyto-sources on total volatile fatty acid production and ammonia-N.

**Conclusion:**

It may be concluded that *L. chinensis, M. malabathricum, L. speciosa, S. cumini*, and *T. chebula* are having potent methane suppressing properties as observed *in vitro* in 24 h. These leaves could be supplemented in the animal diet for reducing methane emission; however, *in vivo* trials are warranted to confirm the methane inhibitory action and optimize the level of supplementation.

## Introduction

Rumen methanogenesis is an integral mechanism associated with complex carbohydrate metabolism for the removal of fermentative gases. The removal of fermentative gases particularly carbon dioxide and hydrogen is prerequisite to keep the redox potential within the threshold limit. On the contrary, rumen methanogenesis invariably contributes to the global warming apart from depriving the host animal to a significant fraction of biological energy. The energy loss due to enteric methane emission varies between 2% and 12% depending on the feed type and their digestibility [1]. The host animal is deprived for 39.5 KJ energy for each liter of enteric methane emission [2]. About 90 Tg enteric methane is added per year to the global atmospheric pool [3], and 11% of the global enteric methane emission is contributed by the Indian livestock [4]. The acute shortage of quality feed and fodders in the country and feeding primarily on high fiber diet are few of the obvious reasons for large methane emission from Indian livestock. Feeding of ruminants on quality feed, i.e., succulent fodder and concentrate is probably the best approach for achieving methane reduction, but factors such as increasing human population, limited land allocation under fodder production, and food-feed-fuel competition pose a check on the use of high-quality feeds for feeding purpose. Further, the use of ionophores such as monensin and lasalocid as feed additives and methane suppressant has been banned in many countries, and others are also in process of following the same.

Therefore, it has become a necessity for the animal nutritionist to search for new feed resources which could be used with dietary ingredients for the purpose of methane mitigation. The anti-methanogenic properties of phyto-sources are usually due to the secondary metabolites which are integral component and serve as first line of defense against insect and pest. Tannins, saponin, and essential oil are few important plant secondary metabolites; however, their anti-methanogenic properties have recently been explored [5-7] and tannin is largely studied plant secondary metabolites among all. However, there has been a variation in tannin’s activity from different sources and all the phyto-sources are not equally effective in achieving the methane reduction even though they possess similar tannin content [6]. Further, the phyto-source available in one geographical location may not be available in farther most located area [6,8,9]. Hence, we should keep broadening the list of phyto-sources that contain measurable tannin content and promising for methane reduction. Diverse kind of phyto-sources is abundant in Northeast state, Assam, which shared Himalayan and Indo-Burmese biodiversity hotspot [10]; however, these sources never been explored for their anti-methanogenic properties.

Thus, the present study was undertaken to investigate the anti-methanogenic potential of 20 medicinal phyto-sources abundantly available in Northeast India.

## Materials and Methods

### Ethical approval

As the study was in vitro and not conducted on live animals; hence, no ethical approval was required for the study.

### Sample collection

A total of 20 phyto-samples (leaves) known for their ethnomedicinal properties were collected from the Northeastern state, Assam (26.2006°N and 92.9376°E), India, during March to April 2014. All the plants were in their reproductive stage (capable of producing reproductive parts). Approximately 500 g of fresh leaves (both mature and immature) were harvested from the tree and air-dried for 10 days. The air-dried samples were brought to the Energy Metabolism Laboratory of the National Institute of Animal Nutrition and Physiology, Bengaluru, and ground samples to 1 mm size using Cyclotec Mill for further analysis. The ground samples were preserved in airtight container and stored after labeling in dark place to prevent the degradation of phenolics.

### Chemical composition

The leaves sample were analyzed in triplicate for crude protein (CP) using standard procedure [11]; while neutral detergent fiber (NDF) and acid detergent fiber (ADF) of leaves were estimated following Van Soest *et al*. [12] methodology. The tannin content in leaves was determined as per a study of Makkar [13]. Briefly, 0.2 g ground sample was extracted in 10 ml aqueous acetone (acetone-water, 7:3) for 20 min in an ultrasonic water bath. The extraction was repeated twice, and the extract was centrifuged (6000 g) for 10 min at 4°C. The supernatant was combined and used for tannin analysis on the same day. Total phenol (TP) and total tannin (TT) in samples were assessed by modified Folin–Ciocalteu method [13] using polyvinylpolypyrrolidone and expressed as gallic acid equivalents. However, condensed tannin (CT) in sample was analyzed by butanol–HCl–iron method [13] and expressed as leucocyanidin equivalents. The hydrolysable tannin (HT) was calculated by difference of TT and CT [6].

### *In vitro* incubation and gas analysis

For *in vitro* studies, rumen liquor was collected at 9.00 h from two cannulated Holstein-Friesian crossbred bulls (330 kg) before morning feeding. The cannulated animals were fed on a total mixed ration comprising finger millet (*Eleusine coracana*) straw and commercial concentrate mixture mixed in the ratio of 70:30. The rumen liquor collected was strained through muslin cloth prior adding to the buffer (bicarbonate, mineral, and distill water) prepared as per Menke *et al*. [14]. This was followed by incubation of air-dried 200 mg sample along with 30 ml buffered rumen inoculum in 100 ml calibrated glass syringe placed in a water bath shaker at 39°C. All samples were incubated 2 consecutive times, wherein three replicates of each samples were used in individual incubation. For every set of incubation and to nullify the effect of rumen inoculum, three syringes were kept as blank (without sample). Individual incubation was set up with three replicates of standard hay and concentrate from Hohenheim University as positive control [14]. Fermentation was terminated on the completion of 24 h. The gas production was calculated by difference of final and initial piston position and considering the gas production from blank syringes which contains no inoculum. The effect of tannin on methane production and rumen fermentation was confirmed by incubating the samples with polyethylene glycol (PEG)-6000 in the ratio of 1:2. Gas samples were collected in evacuated glass vials and 1 mL was withdrawn with the help of Hamilton Syringe for the gas chromatograph analysis. Methane was estimated using gas chromatograph (Chemito GC-1000, India) equipped with thermal conductivity detector and Porapak-Q column. Injector, column, and detector temperatures were kept 60°, 100°, and 110°C, respectively. Methane standard samples (22.4%, Chemix Specialty Gases and Equipment, Bengaluru, India) were run before and after analysis of the test samples. Based on the area and percentage of the standard gas, the percentage of methane in test sample was calculated using the following formula:

CH4 (%)=((standard CH4 concentration)×(area of test sample))/(area of standard CH4)

### Rumen fermentation characteristics

On the termination of incubation (24 h), the buffered rumen fluid was transferred into ice-cold graduated tube to stop further fermentation and pH was measured (Eutech Instruments, pH meter Cyberscan) immediately. The samples were, later on, stored at −20°C until further analysis. Stored rumen liquor samples were thawed and used for analyzing ammonia-N [15] by taking 1 mL of buffered rumen liquor in the outer chamber of the dish, 1 mL boric acid indicator in the central chamber, and 1 mL 45% potassium carbonate solution to the opposite side of rumen fluid. The content was mixed and titrated against standard sulfuric acid. Total volatile fatty acid (TVFA) in the samples was estimated according to Barnett and Reid [16]. In brief, 1 mL rumen fluid with an equal volume of buffer (potassium oxalate + oxalic acid) was taken in Markham apparatus, and distillate was collected in a flask placed in ice bath. Few drops of phenolphthalein indicator were added to 100 mL distillate and then titrated against alkali.

### Rumen protozoa enumeration

Incubation fluid for the enumeration of protozoa was preserved by adding formaldehyde solution (37-41%) in 1:2. The rumen protozoa were enumerated with the help of counting chamber having a depth of 0.1 mm. The protozoa numbers were calculated according to the study of Kamra *et al*. [17]. Rumen protozoa based on their morphological characteristics were categorized and were identified to generic level and classified into small spirotrichs mainly entodinimorphs (with an average size of 42 µm × 23 µm) and large spirotrichs, i.e., diplodinia (average size of 132 µm × 9 66 µm).

### Statistical analysis

Data were analyzed using SPSS [18] version 20.0 software package. The effect of sample and sample with PEG addition on various parameters was analyzed in repeated measures ANOVA. The repeated effect is the number of observations/replicates and incubation. Results were presented as means and standard error of means. The significance was checked by comparing mean values using Tukey’s test.

## Results

The nutrient composition and phenol content of the leaf samples known for ethnomedicinal properties are presented in Table-1. The CP content of the test phyto-sources ranged between 62 and 198 g/Kg dry matter (DM). The CP content in *Leucas linifolia* was highest (198), while *Michelia champaca* reported with lowest CP (62). Neutral detergent fiber (NDF) varied (p<0.05) among the phyto-sources. *Litchi chinensis* had highest NDF (693), while *Citrus grandis* had the lowest (295). Similarly, the ADF content also varied (p<0.05) among the samples. The TP content of the phyto-sources varied considerably and reported as high as 211 g/kg DM (*Syzygium cumini)* and low as 11 g/Kg DM (*Ficus hispida*). Leaves from *Terminalia chebula, S. cumini*, and *Melastoma malabathricum* possess highest tannin content (>100 g/Kg DM); however, the leaves from *F. hispida* and *Alstonia scholaris* had negligible tannin content. The CT varied between 0.24 (*L. linifolia*) and 50.4 g/Kg DM (*Machilus bombycina*). Among the studied samples, *S. cumini, M. malabathricum*, and *T. chebula* are high HT-containing sources, while *Flacourtia jangomos, Lagerstroemia speciosa*, and *Christella parasitica* (L.) Lev have medium HT content and *F. hispida, Elaeocarpus floribundus, Mallotus ferrugineus, C. grandis* (L.) osb.*, A. scholaris, M. champaca, Aquilaria malaccensis, Antidesma ghaesembilla*, and *L. linifolia* have very low HT content. On the other hand, *M. bombycina* is a potent source of CT, whereas *Achras zapota, L. chinensis, Baccaurea ramiflora*, and *Mesua ferre*a contain good proportion of both CT and HT. *A. malaccensis* and *A. ghaesembilla* are the low CT-containing sources.

**Table-1:** Nutrient content and phenol content of medicinal plant leaves (g/kg DM) from Northeast state, Assam.

Common name	Scientific name	CP^1^	NDF^1^	ADF^1^	TP^2^	TT^2a^	CT^2b^	HT^2c^
Jarul	*Lagerstroemia speciosa*	119^gh^	668^b^	535^b^	57.7^i^	49.8^h^	1.15^q^	48.6^f^
Panpatti	*Christella parasitica* (L.) Lev.	143^de^	378^i^	257^j^	65.8^gh^	50.6^h^	3.68^m^	46.9^f^
Droton	*Leucas linifolia*	198^a^	338^k^	230^k^	22.0^l^	12.5^k^	0.24^r^	12.3^i^
Hairy fig	*Ficus hispida*	115^h^	519^d^	396^e^	11.0^n^	7.13^l^	1.24^q^	5.88^jk^
Black currant	*Antidesma ghaesembilla*	187^b^	488^e^	470^c^	20.3^l^	16.9^j^	10.3^i^	6.67^jk^
Indian olive	*Elaeocarpus floribundus*	163^c^	384^hi^	251^j^	26.5^k^	24.0^i^	4.51^l^	19.5^h^
Jamun	*Syzygium cumini*	78^j^	405^g^	379^f^	211^a^	190^a^	23.9^f^	166^a^
Burmese grape	*Baccaurea ramiflora*	68^k^	321^l^	259^j^	94.8^f^	86.2^e^	38.8^d^	47.5^f^
Lichee	*Litchi chinensis*	97^i^	693^a^	612^a^	68.6^g^	61.4^f^	39.4^c^	22.0^h^
Rusty kamala	*Mallotus ferrugineus*	187^b^	450^f^	384^ef^	12.4^mn^	9.25^l^	1.24^q^	8.00^j^
Indian rose chestnut	*Mesua ferrea*	78^j^	560^c^	416^d^	64.7^h^	57.5^g^	30.9^e^	26.6^g^
Singapore rhododendron	*Melastoma malabathricum*	131^f^	364^j^	258^j^	124^c^	118^c^	9.14^j^	109^c^
Coffee plum	*Flacourtia jangomos*	92^i^	354^j^	211^l^	121^d^	97.7^d^	5.16^k^	92.5^d^
Pomelo	*Citrus grandis (L.) osb.*	160^c^	295^m^	263^ij^	14.1^mn^	8.52^l^	1.79^p^	6.72^jk^
Som	*Machilus bombycina*	123^g^	521^d^	469^c^	62.7^h^	54.4^g^	50.4^a^	3.97^k^
Chiko	*Achras zapota*	69^k^	462^f^	337^g^	108^e^	96.8^d^	44.4^b^	52.3^e^
Agarwood	*Aquilaria malaccensis*	145^d^	394^gh^	273^i^	34.6^j^	26.0^i^	10.8^g^	15.2^i^
ChebulicMyrobalan	*Terminalia chebula*	145^d^	453^f^	309^h^	161^b^	154^b^	10.5^h^	144^b^
Devil tree	*Alstonia scholaris*	138^e^	393^gh^	211^l^	12.1^n^	7.55^l^	1.97^o^	5.58^jk^
Golden champaca	*Michelia champaca*	62^l^	492^e^	413^d^	15.3^m^	9.55^kl^	3.39^n^	6.17^jk^
SEM		4.63	11.8	12.5	5.01	4.70	1.49	4.34
p value		<0.001	<0.001	<0.001	<0.001	<0.001	<0.001	<0.001

CP=Crude Protein, NDF=Neutral detergent fiber, ADF=Acid detergent fiber, TP=Total phenol, TT=Total tannin, CT=Condensed tannins, HT=Hydrolysable tannins. ^1^ Each value is the mean of four observations. ^2^ Each value is the mean of six observations, ^a^ As tannic acid equivalent, ^b^ As leucocyanidin equivalent, ^c^ Difference of a and b. SEM=Standard error of mean, DM=Dry matter. Mean value bearing different superscript in a column are significant (p<0.05)

### Total gas and methane production

Data pertaining to the effect of different leaves and PEG addition on total gas and methane production are presented in Table-2. Results indicated statistically significant (p<0.001) variation in total gas production among the studied samples. *C. parasitica* produced maximum gas (33.3 ml/200 mg DM) on the incubation, while *Lagerstroemia speciosa*, *M. malabathricum, T. chebula*, and *L. chinensis*, on the other hand, produced very less total gas. The effect of CT-containing sources such as *L. chinensis* and *M. bombycina* on total gas production was comparable (10.0-16.7 ml/200 mg DM) to the HT sources such as *T. chebula* and *S. cumini*. However, the phyto-sources having appreciable concentration of both CT and HT (*M. ferrea* and *A. zapota*) showed comparatively less impact on gas production than either CT or HT. The adverse effect of tannin on gas production was attenuated with PEG-6000 addition, and a significant change (p<0.001) in gas production was noticed. The increase in gas production due to the inclusion of PEG as represented by tannin bioassay (TBA) was highest in *M. malabathricum* (128) followed by *L. speciosa* (100) (Table-2). Among the samples, *L. chinensis, M. malabathricum, L. speciosa, S. cumini*, and *T. chebula* produced lowest methane (ml/200 mg DM), while *C. parasitica, C. grandis, L. linifolia*, and *A. malaccensis* produced comparatively more methane. The methane production from the phyto-sources-containing HT was lesser than those with high CT content.

**Table-2:** Effect of PEG-6000 addition on *in vitro* total gas and methane production (ml/200 mg DM) from medicinal plant leaves.

Scientific name	Total^#^ gas (ml/200 mg DM)*		TBA*	Methane (ml/200 mg DM)*		Methane reduction per ml of total gas reduction*
	
	−PEG	+PEG		−PEG	+PEG	
*Lagerstroemia speciosa*	10.4^i^	20.8^f^	100^ab^	1.44^ghi^	2.96^hi^	0.14^e^
*Christella parasitica (L.) Lev.*	33.3^a^	34.9^b^	5.00^f^	5.84^a^	6.34^d^	0.30^cde^
*Leucas linifolia*	28.2^b^	30.5^d^	8.40^f^	5.40^ab^	6.68^cd^	0.65^a^
*Ficus hispida*	15.9^gh^	29.4^d^	84.8^bc^	1.78^ghi^	4.50^ef^	0.20^e^
*Antidesma ghaesembilla*	14.0^h^	15.3^h^	9.60^f^	1.92^gh^	2.28^hi^	0.28^cde^
*Elaeocarpus floribundus*	17.6^efg^	20.6^f^	18.0^ef^	3.06^ef^	4.70^ef^	0.58^abc^
*Syzygium cumini*	16.1^g^	20.8^f^	30.4^ef^	1.42^ghi^	4.10^fg^	0.63^ab^
*Baccaurea ramiflora*	18.6^def^	30.9^d^	66.8^cd^	4.46^cd^	7.54^bc^	0.25^e^
*Litchi chinensis*	10.0^i^	16.1^g^	61.6^cd^	1.04^i^	2.12^i^	0.18^e^
*Mallotus ferrugineus*	16.0^g^	18.2^g^	13.8^ef^	1.80^ghi^	2.72^hi^	0.41^abcde^
*Mesua ferrea*	19.3^de^	34.9^b^	80.6^bc^	3.12^e^	8.00^b^	0.31^cde^
*Melastoma malabathricum*	10.8^i^	24.2^e^	128^a^	1.12^hi^	3.20^gh^	0.16^e^
*Flacourtia jangomos*	24.7^c^	30.4^d^	23.2^ef^	4.88^bcd^	7.04^bcd^	0.38^abcde^
*Citrus grandis (L.) osb.*	27.8^b^	36.9^a^	32.6^ef^	5.52^ab^	10.8a	0.58^abc^
*Machilus bombycina*	16.7^fg^	22.0^f^	32.4^ef^	2.12^g^	5.14^ef^	0.58^abcd^
*Achras zapota*	20.6^d^	37.7^a^	83.0^bc^	2.24^fg^	8.08^b^	0.34^bcde^
*Aquilaria malaccensis*	29.4^b^	34.2^c^	16.4^ef^	5.24^abc^	6.52^cd^	0.27^de^
*Terminalia chebula*	10.4^i^	14.4^h^	38.6^de^	1.82^ghi^	2.96^hi^	0.28^cde^
*Alstonia scholaris*	24.1^c^	29.4^d^	22.0^ef^	4.24^d^	6.56^cd^	0.44^abcde^
*Michelia champaca*	18.3^ef^	20.8^f^	13.6^ef^	4.24^d^	5.18^e^	0.39^abcde^
SEM	0.67	0.75	3.67	0.17	0.23	0.02
p value	<0.001	<0.001	<0.001	<0.001	<0.001	<0.001

PEG=Polyethylene glycol, TBA=Tannin bioassay, SEM=Standard error of mean.

* Each value is the mean of five observations. Mean value bearing different superscript in a column are significant (p<0.05).

# Total gas=(Plunger position at 24 h−plunger position at 0 h=x ml-average of gas produced in blank syringe=Total gas/200 mg of sample, which is converted to the DM content of sample, thus total gas/200 mg DM). DM=Dry matter

### Fermentation characteristics

Rumen pH was affected (p<0.05) with incubation source; however, no notable depression in ruminal pH was observed below 6.47. Similarly, TVFA production also varied among the samples. Results demonstrated that CT-containing sources such as *M. bombycina* and *L. chinensis* and promising HT sources such as *T. chebula* and *S. cumini* have less effect on TVFA production, while a few HT sources such as *M. malabathricum* and *L. speciosa* adversely affect TVFA production to the maximum extent (Table-3). PEG-6000 addition shows an increase in TVFA production (p<0.001). Tannin from the leaves also affected rumen ammonia-N significantly (p<0.001). Sources such as *T. chebula, A. scholaris*, and *M. champaca* lower ammonia (p<0.05) production. The adverse impact of tannin on ammonia-N was attenuated with PEG (p<0.001) addition. Samples rich in HT content (*S. cumini, M. malabathricum*, and *E. floribundus*) show highest increase in percentage of ammonia-N as compared to the rest of the samples on PEG inclusion in our study.

**Table-3:** Effect of medicinal plant leaves and PEG addition on rumen fermentation characteristics.

Common name	pH*		TVFA (mmol/dL)*		Ammonia nitrogen (mg/dL)*	
		
	−PEG	+PEG	−PEG	+PEG	−PEG	+PEG
*Lagerstroemia speciosa*	7.03^ab^	7.04^ab^	8.50^f^	9.23^l^	25.9^ab^	27.8^ab^
*Christella parasitica* (L.) Lev.	6.73^gh^	6.77^h^	11.8^bc^	14.6^ab^	27.5^a^	29.6^a^
*Leucas linifolia*	6.96^bcd^	6.97^abcd^	12.0^abc^	13.4^de^	19.8^defg^	22.2^efg^
*Ficus hispida*	6.47^i^	6.50^i^	10.1^de^	11.2^ijk^	23.3^bc^	25.9^bcd^
*Antidesma ghaesembilla*	6.89^de^	7.02^abc^	12.5^ab^	13.4^def^	21.0^cde^	27.8^ab^
*Elaeocarpus floribundus*	6.99^abc^	7.03^abc^	10.1^de^	11.7^hi^	18.2^fgh^	25.0^cd^
*Syzygium cumini*	7.05^a^	7.06^a^	10.1^de^	10.7^kj^	20.3^defg^	25.9^bcd^
*Baccaurea ramiflora*	6.78^fg^	6.88^efg^	12.0^abc^	13.7^cd^	15.6^h^	19.8^g^
*Litchi chinensis*	6.92^cd^	6.95^cde^	12.1^abc^	12.7^fg^	25.7^ab^	27.3^abc^
*Mallotus ferrugineus*	6.88^de^	6.89^defg^	13.0^ab^	14.9^a^	21.2^cde^	21.5^fg^
*Mesua ferrea*	6.90^de^	6.92^de^	11.8^bc^	14.8^ab^	21.7^cd^	25.0^cd^
*Melastoma malabathricum*	7.01^ab^	7.04^ab^	9.77^e^	10.5^k^	17.7^gh^	24.0^def^
*Flacourtia jangomos*	6.74^gh^	6.75^h^	12.1^abc^	13.5^d^	19.8^defg^	20.8^g^
*Citrus grandis* (L.) osb.	6.89^de^	6.91^def^	11.1^cd^	12.1^gh^	26.1^a^	27.8^ab^
*Machilus bombycina*	6.95^bcd^	6.96^bcde^	13.0^ab^	14.2^bc^	22.4^cd^	24.0^def^
*Achras zapota*	6.75^fgh^	6.76^h^	10.1^de^	10.7^kj^	20.5^def^	24.3^de^
*Aquilaria malaccensis*	6.68^h^	6.83^fgh^	12.1^abc^	12.8^ef^	18.7^efg^	21.5^fg^
*Terminalia chebula*	6.83^ef^	6.90^defg^	13.1^a^	13.6^cd^	7.70^j^	8.38^i^
*Alstonia scholaris*	6.91^cde^	7.06^a^	10.6^de^	12.0^h^	6.53^j^	7.47^i^
*Michelia champaca*	6.75^fgh^	6.81^gh^	10.1^de^	11.2^ij^	10.7^i^	11.9^h^
SEM	0.01	0.01	0.12	0.15	0.53	0.57
p value	<0.001	<0.001	<0.001	<0.001	<0.001	<0.001

PEG=Polyethylene glycol, TVFA=Total volatile fatty acids, SEM=Standard error of mean.

* Each value is the mean of six observations. Mean value bearing different superscript in a column are significant (p<0.05)

### Effect on rumen protozoa population

Total protozoa numbers (×10^5^) varied (p<0.05) considerably (0.122-0.262) among the phyto-sources (Table-4). *C. parasitica, T. chebula*, and *M. bombycina* exhibited maximum influence on rumen protozoa population. Phyto-sources such as *M. malabathricum* and *F. jangomos* that contain high HT did not affect rumen protozoa. Similarly, the effect of *M. ferrea*, a good source of both HT and CT, also did not affect the ciliate population. PEG addition to the basal diet increased (p<0.05) the protozoal numbers in comparison of their respective control. In this study, entodinia were found major ciliates (Table-4); however, their numbers also varied (p<0.05) among the studied samples.

**Table-4:** Effect of medicinal plant leaves on rumen protozoal population.

Common name	Entodina (×10^5^)*		Holotricha (×10^5^)*		Total protozoa (×10^5^)*		% increase*
		
	−PEG	+PEG	−PEG	+PEG	−PEG	+PEG		
*Lagerstroemia speciosa*	0.176^def^	0.185^cdefg^	0.003^cd^	0.006^cd^	0.178^cd^	0.191^efghij^	6.84^cde^
*Christella parasitica* (L.) Lev.	0.125^i^	0.157^ghij^	0.003^cd^	0.017^ab^	0.128^fg^	0.174^hijk^	37.5^ab^
*Leucas linifolia*	0.183^cdef^	0.220^c^	0.003^cd^	0.008^bcd^	0.187^bc^	0.228^cd^	23.0^bcd^
*Ficus hispida*	0.192^bcd^	0.212^cd^	0.001^d^	0.002^cd^	0.193^bc^	0.214^cdef^	10.8^bcde^
*Antidesma ghaesembilla*	0.173^def^	0.172^efghi^	0.001^d^	0.004^cd^	0.173^cd^	0.175^ghijk^	1.44^de^
*Elaeocarpus floribundus*	0.206^bc^	0.180^defghi^	0.002^cd^	0.001^d^	0.208^b^	0.181^fghijk^	0.00^e^
*Syzygium cumini*	0.160^efgh^	0.206^cde^	0.004^bcd^	0.006^cd^	0.165^cde^	0.211^cdefg^	31.3^bc^
*Baccaurea ramiflora*	0.165^defg^	0.179^defghi^	0.001^d^	0.002^cd^	0.166^cde^	0.181^fghijk^	10.4^bcde^
*Litchi chinensis*	0.184^cde^	0.207^cde^	0.002^cd^	0.003^cd^	0.186^bc^	0.211^cdefgh^	13.5^bcde^
*Mallotus ferrugineus*	0.191^bcd^	0.197^cdef^	0.000^d^	0.001^cd^	0.192^bc^	0.198^defghi^	3.70^cde^
*Mesua ferrea*	0.210^bc^	0.211^cd^	0.000^d^	0.007^bcd^	0.210^b^	0.219^cde^	4.26^cde^
*Melastoma malabathricum*	0.213^b^	0.211^cd^	0.001^d^	0.001^cd^	0.214^b^	0.242^c^	13.1^bcde^
*Flacourtia jangomos*	0.251^a^	0.279^b^	0.000^d^	0.001^d^	0.251^a^	0.280^b^	11.7^bcde^
*Citrus grandis* (L.) osb.	0.260^a^	0.429^a^	0.001^cd^	0.000^d^	0.262^a^	0.429^a^	64.0^a^
*Machilus bombycina*	0.132^hi^	0.131^j^	0.007^bc^	0.017^ab^	0.139^efg^	0.148^k^	7.88^cde^
*Achras zapota*	0.139^ghi^	0.147^hij^	0.017^a^	0.026^a^	0.156^def^	0.172^ijk^	10.5^bcde^
*Aquilaria malaccensis*	0.155^fgh^	0.162^fghij^	0.001^cd^	0.001^cd^	0.156^def^	0.163^ijk^	4.68^cde^
*Terminalia chebula*	0.120^i^	0.150^ghij^	0.001^cd^	0.004^cd^	0.122^g^	0.154^jk^	26.7^bcd^
*Alstonia scholaris*	0.141^ghi^	0.145^ij^	0.010^b^	0.011^bc^	0.151^defg^	0.155^jk^	3.02^cde^
*Michelia champaca*	0.172^def^	0.183^defgh^	0.001^d^	0.001^d^	0.172^cd^	0.184^efghijk^	6.76^cde^
SEM	0.004	0.002	0.000	0.001	0.002	0.006	1.96
p value	<0.001	<.001	<0.001	<0.001	<0.001	<0.001	<0.001

PEG=Polyethylene glycol, SEM=Standard error of mean.

* Each value is the mean of five observations; Mean value bearing different superscript in a column are significant (p<0.05). A percentage increase represents increase with PEG addition compared to without PEG. Spirotrichs (mainly diplodinia with an average size of 132 mm×66 mm). Spirotrichs not identified to generic level were classified into small spirotrichs (mainly entodina with an average size 42 mm×23 mm) and large spirotrichs (mainly diplodinia with an average size of 132 mm×66 mm)

## Discussion

Northeast India from where phyto-sources used in this study were collected forms a unique biogeographic province in the world by sharing a part of both Himalayan and Indo-Burmese biodiversity hotspots [10]. The phyto-sources were evaluated *in vitro* for their anti-methanogenic properties to supplement the knowledge and broaden the list of feed additives which induces positive changes by altering rumen fermentation to achieve significant methane reduction. Among the studied leaves, *C. parasitica* produced maximum *in vitro* gas, while *L. speciosa, T. chebula*, *L. chinensis*, and *M. malabathricum* produced minimum. To ascertain the effect of tannin on gas production, PEG-6000 was used in our study. PEG-6000 neutralizes the effect of tannin by forming tannin-PEG complex and thereby increases carbohydrate digestibility [19,20]. In this study, wide variation in gas production was seen among the phyto-sources and the addition of PEG-6000 has shown almost double-fold increase in the gas production, suggesting the influence of tannin on carbohydrates. However, PEG addition to the basal diet always not increased fermentation of the tannins-containing basal diet. This unresponsive mechanism of PEG towards tannins could be attributed to the less bacterial adhesion to the feed because of PEG [21]. Tannins (both CT and HT) interact with carbohydrate by hydrogen bonding and hydrophobic interaction due to its large number of hydroxyl group [22,23]. However, difference in tannin polymer size is one of the main factors affecting its ability to bind to fiber [24], which influences fermentation of tannin-containing phyto-sources. The high tannin content of *T. chebula* and *M. malabathricum* produced relatively low gas than *L. linifolia* and *A. scholaris* which contained comparatively low tannin [25]. These results clearly indicated that tannin level affects the feed fermentation and subsequent gas production. The difference in gas production among the phyto-sources could also be due to the presence of prodelphidin. CT with larger proportion of prodelphidin has high affinity toward fiber as prodelphidin has more hydrogen bonding sites, thus creating a CT-fiber complex which reduces fiber degradation and affects gas production differently [26]. In our study, we have not estimated prodelphidin; however, there presence in tannin-containing phyto-sources might be having some effect on the gas production. Apart from the tannin content, non-starch polysaccharide (NSP) [27], fiber fraction, and lignin content also play important role in influencing gas production [28]. Karabulut *et al*. [29], in an *in vitro* study, also reported a significant variation in gas production in different legume hays which is attributed to the variable fiber fractions (NDF and ADF). Variation in cell wall content (i.e., NDF and ADF) among different phyto-sources as observed in this study might reduce the availability of rapidly fermented carbohydrates and thereby suppress the microbial activity, resulting in lower gas production. However, it was also found that, with the same carbohydrate content, different gas values can be recorded due to difference in microbial protein synthesis [28]. Studies have also shown that difference in gas production was recorded despite similar tannin and NSP concentration in a study carried out on two *Leucaena* species (*L. leucocephala* and *L. pallida*) *in vitro* [27]. They stated that this difference could be due to difference in tannin chemistry (molecular size and monomer composition). The different species of phyto-source also determine the ability of fiber to complex with polyphenols, thereby affecting the gas production as observed in a study carried out using 17 Zimbabwean browse species [30]. Hence, the effect of phyto-sources on gas production is an influence of many factors such as its variable tannin, NDF, and ADF content. Furthermore, the variation in tannin polymer size, lignin, NSP, and prodelphidin content which were not estimated in this study might have influenced the gas production. Thus, phyto-sources such as *S. cumini* even with quite large tannin content could not produce less gas as expected.

The effect of tannin-containing phyto-sources on methane production depends largely on fermentation of these phyto-sources, bacteriocidal and bacteriostatic effects of tannin on rumen microbes, and rumen enzymes [25]. The variable tannin biological properties depend on tannin chemical structure and degree of polymerization [31]. Results from our studies revealed variable efficacy of different phyto-sources for methane reduction. This could be attributed to the variable tannin content and tannin structural composition [24] among the studied sources, even though the structural analysis was not carried out in our study. Contrary to high tannin sources such as *S. cumini* and *T. chebula*, low methane production was observed in *M. malabathricum* and *L. chinensis*. This could be due to the presence of other secondary metabolites which might be having additive effect with tannin on methane reduction. For instance, the presence of flavonoids [32], alkaloids, and saponin glycosides [33] which has antimicrobial properties [34,35] in *L. chinensis* might be responsible for reducing methane along with tannin. Nevertheless, methanogens were not quantified in our study, but it is well-established fact that tannin inhibits methanogenesis directly through methanogen reduction or indirectly through antiprotozoal action, thereby restricting H_2_ transfer to methanogen [8]. The reduction in methane production by *S. cumini*, *T. chebula*, and *L. chinensis* was supported by a reduced protozoa numbers justifying the indirect effect of tannin on protozoa leading to less methane production. Furthermore, tannins often exhibit a reduction in degradation of nutrients and alter the site of degradation post-ruminally. The study of Hariadi *et al*. [36] strengthened our results, who reported that tannin content had negative correlation with methane production. Our results demonstrated that *C. parasitica* (L.) and *F. jangomos* in spite of their high tannin content produced comparatively more methane which could be due to the heterogeneous nature of tannin and a variable degree of polymerization that exerts different biological activities. However, methane production was low from the phyto-sources such as *S. cumini* and *T. chebua-*containing high HT. Jayanegara *et al*. [37] also reported that HT has greater ability to reduce methane than CT. Addition of PEG in this study attenuated the adverse impact of tannin on methane production than their corresponding control. Among the studied phyto-sources, *L. linifolia* (0.65*), S. cumin*i (0.63), *E. flribundus* (0.58*), M. bombycina* (0.58), an*d C. grandis* (L.) (0.58) were more effective in reducing methane (ml/ ml of reduction in total gas). The screening of the promising sources for further *in vivo* evaluation in ruminants before recommending the inclusion level is a big challenge and the criteria should be the reduction in methane per ml of reduction in total gas production [6]. The sources which have the highest ratio in methane reduction with per unit of gas reduction should be selected for optimizing their level of inclusion in diet and for *in vivo* evaluation.

The effect of tannins from different sources on rumen pH remained inconsistent. However, in our study, a significant impact of tannin on pH was observed which could be attributed to the different soluble carbohydrate content of studied phyto-sources. Previous studies [38,39] also reported that soluble carbohydrate has a well-defined adverse impact on rumen pH. Dschaak *et al*. [40] studied the effect of CT supplementation on rumen fermentation and found no significant effect of tannin on rumen pH. He suggested the significance of particle size of the diet and its distribution apart from soluble carbohydrate content role in influencing rumen pH.

The adverse impact of high tannin content on feed fermentation is well known where it affects the degradation of fiber in rumen and hence the nutrient availability. There is a wide variation observed in TVFA production from 200 mg of substrate. These results are similar to the study carried out by Bhatta *et al*. [6,8], where numerous tropical phyto-sources were tested for its methane reduction potential. Phyto-sources such as *M. malabathricum* and *L. speciosa* having high tannin content show significant effect on gas production as well as TVFA. The significant decrease in TVFA production from the phyto-sources appeared an aggregate result of high structural carbohydrate and tannin content. The TVFA which is produced due to the fermentation of feedstuff is mainly constituted by acetate and butyrate, with propionate yielding gas only due to buffering of the acid [41]. The high fiber as well as tannin content or both decreased the fermentability of phyto-sources as evidenced from decreased volatile fatty acid production [25]. However, feeds that produce high amounts of propionate yield lower gas volumes [42]. The presence of non-fiber carbohydrate also contributes to more propionate production [41]. The higher proportion of propionate production leads to a weaker correlation between gas and VFA production [41]. Increase in propionate production is a competitive pathway for methane and thus lowers methane production [43]. In case of *T. chebula*, decrease in methane is evident, so it can be assumed that propionate is increased here. Apart from that, there is a decrease in protozoa population recorded in *T*. *chebula*. These rumen protozoa ingest starch granules and decrease starch degradation [44]. Hence, in *T. chebula*, more starch degradation occurs and thus contributes to increases the TVFA content. Similar effect was recorded by Santra and Karim [44] where defaunation effect shows increase in TVFA production. The addition of PEG shows improvement in TVFA production as compared to their corresponding control, indicating the impact of tannin on TVFA production. The different abilities of tannin’s phenolic group to bind with PEG as reported earlier [20] could be the reason for variable increase in TVFA production.

Tanniniferous phyto-sources have a most pronounced effect on ammonia-N [45], and it was confirmed in the present study too where PEG addition improved the ammonia-N concentration in test treatments as compared to the respective control. Tannins have high affinity toward protein and form cross-linkages and therefore decrease the protein degradation in rumen and increase the post-ruminal utilization. Bhatta *et al*. [46], in an *in vitro* study, reported the alteration in nitrogen excretion more from urinary-N to fecal nitrogen and observed an overall improvement in nitrogen utilization due to post-ruminal degradation. Our study recorded similar trend with Bhatta *et al*. [6], in ammonia-N with 200 mg of incubated sample proving the effect of tannin in reducing ruminal ammonia-N production. The phyto-sources which have both CT and HT content in them such as *A. zapota, M. feea, B. ramiflora, A. ghaesembilla*, and *A. malaccensis* produce more variation in ammonia-N. This huge variation in CT- and HT-containing phyto-sources is due to partially reversible nature of protein-HT complex [6] as rumen bacteria can dissociate protein-HT complex easily than protein-CT complexes. On the other hand, samples which are having more HT content such as *S. cumini, L. speciosa*, and *C. parasitica* (L.) Lev have ammonia-N concentration in a narrow range of (20.3-27.5 mg/dl). However, an interesting result is observed in case of *T. chebula* and *S. cumini* which are both a rich source of HT. The former one produced very low ammonia-N as compared to the later. This low concentration of ammonia-N in *T. chebula* compared to *S. cumini* could be due to less number of protozoa in the former. This leads to decrease in bacterial protein breakdown and eventually feed protein degradability [47]. The low protozoa number of *T. chebula* was also observed in Bhatta *et al*. [8] study. Deaville *et al*. [48], in a study, reported that the binding strength of tannin-protein complex depends on the polyol core of the HT and also stated that the loss of conformational freedom in tannin structure weakens its bond to protein. Since in this study exact structural elucidation is not done, we cannot assure this, but the difference in the binding ability of HT to protein could also be the reason behind wide variations in ammonia-N content as weakly bound proteins will be easy to degrade and resulting in more ammonia-N than the tightly bound ones. The PEG–tannin interaction showed an increase in ammonia-N production up to 35% in few of the HT sources, which possibly was due to more hydroxyl (OH) group in HT than CT sources. González *et al*. [49] had carried out a study where they found that chestnut tannins were more effective in protecting soybean meal than quebracho tannins from *in vitro* bacterial degradation because of the presence of more OH group than quebracho tannin as OH group forms hydrogen bond with NH_2_, SH, OH, and CO groups of proteins.

Protozoa are well known for the interspecies hydrogen transfer to the archaea which use it for the reduction of CO_2_ into methane [47]. The PEG addition showed an increase in the total protozoa; however, the increase in the total protozoa was not similar across the studied phyto-sources. This variation in protozoal action could be attributed to the variable efficiency of PEG to bind tannin from different sources as well as the structural variation of tannin. In our study, entodinomorphs were major ciliates while holotrichs represented the small fraction of total protozoa irrespective of the phyto-sources. Bhatta *et al*. [8] also reported that holotrichs were not major group of the rumen protozoa. Previous studies [50] on HT have shown a significant effect on entodina and total protozoa population. In agreement to their results, we have observed a significant effect of *S. cumini* and *T. chebula* which are promising HT source on rumen protozoa whose effect is counteracted by PEG addition showing an increase in total protozoa population up to 31.4%. While in *M. malabathricum*, which is a good tannin source, addition of PEG had increase protozoa population only by 13.1%. A study carried out by Mamat *et al*. [51] on *M. malabathricum* reported the presence of other secondary metabolites such as saponins and flavonoids. Both flavonoid [52,53] and saponins are known to reduce ciliate population [54,55]. Since PEG-6000 is a tannin binder [19], it complexes only with tannin, leaving the flavonoids and saponin [51]. Thus, the expected increase in protozoa population was not observed. Similarly, *F. jangomos* and *L. speciosa* have shown to impact rumen protozoa, but PEG addition has shown very minimal effect on protozoa reversal to its equilibrium showing the presence of other secondary metabolites like saponin which is known for having negative effect on rumen protozoa count.

## Conclusion

From this study, it can be concluded that *S. cumini, M. malabathricum*, and *T. chebula* are high HT-containing sources, while *F. jangomos, L. speciosa*, and *C. parasitica* (L.) Lev are medium HT-containing sources. On the other hand, *M. bombycina* is a potent source of CT, whereas *A. zapota, L. chinensis, B. ramiflora*, and *M. ferrea* contain good proportion of both CT and HT. Among these phyto-sources, *L. chinensis, M. malabathricum, L. speciosa, S. cumini*, and *T. chebula* are having potent methane suppressing properties as evidenced by lowest methane production when incubated for 24 h as compared to the rest of the samples. Further studies on determination of optimum dose of the above samples are required which can be used to carry out *in vivo* studies so as to establish them as methane-suppressant phyto-source in the ruminant diets.

## Authors’ Contributions

RB and PKM planned and designed the study. The experiment was conducted by LB. APK, AD, PKM, and RB gave necessary inputs during the experimental phases and also allowed the use of instruments for smooth running of the experiment. RB and PKM carried out interpretation of results. LB and PKM participated in data analysis and also involved in the preparation of a draft of the manuscript. All authors read and approved the final manuscript.

## References

[1] Johnson K.A, Johnson D.E (1995). Methane emissions from cattle. J. Anim. Sci.

[2] Guan H, Wittenberg K.M, Ominski K.H, Krause D.O (2006). Efficacy of ionophores in cattle diets for mitigation of enteric methane. J. Anim. Sci.

[3] Malik P.K, Bhatta R, Soren N.M, Sejian V, Mech A, Prasad K.S, Prasad C.S (2015). Feed Based Approaches in Enteric Methane Amelioration. Livestock Production and Climate Change.

[4] Malik P.K, Kolte A.P, Dhali A, Sejian V, Thirumalaisamy G, Gupta R, Bhatta R (2016). GHG emissions from Livestock:Challenges and ameliorative measures to counter adversity. In Greenhouse Gases-Selected Case Studies.

[5] Guo Y.Q, Liu J.X, Lu Y, Zhu W.Y, Denman S.E, McSweeney C.S (2008). Effect of tea saponin on methanogenesis, microbial community structure and expression of *mcrA* gene, in cultures of rumen micro-organisms. Lett. Appl. Microbiol.

[6] Bhatta R, Saravanan M, Baruah L, Sampath K.T (2012). Nutrient content *in vitro* ruminal fermentation characteristics and methane reduction potential of tropical tannin-containing leaves. J. Sci. Food Agric.

[7] Patra A.K, Yu Z (2012). Effects of essential oils on methane production and fermentation by, and abundance and diversity of, rumen microbial populations. Appl. Environ. Microbiol.

[8] Bhatta R, Baruah L, Saravanan M, Suresh K.P, Sampath K.T (2013). Effect of medicinal and aromatic plants on rumen fermentation, protozoa population and methanogenesis *in vitro*. J. Anim. Physiol. Anim. Nutr.

[9] Pal K, Patra A.K, Sahoo A (2015). Evaluation of feeds from tropical origin for *in vitro* methane production potential and rumen fermentation *in vitro*. Spanian J. Agric. Res.

[10] Chatterjee S, Saikia A, Dutta P, Ghosh D, Pangging G, Goswami A.K (2006). Biodiversity Significance of North East India.

[11] AOAC (1995). Official Methods of Analysis.

[12] Van Soest P.V, Robertson J.B, Lewis B.A (1991). Methods for dietary fiber, neutral detergent fiber, and nonstarch polysaccharides in relation to animal nutrition. J. Dairy Sci.

[13] Makkar H.P (2003). Quantification of tannins in tree and shrub foliage:A laboratory manual.

[14] Menke K.H, Raab L, Salewski A, Steingass H, Fritz D, Schneider W (1979). The estimation of the digestibility and metabolizable energy content of ruminant feeding stuffs from the gas production when they are incubated with rumen liquor *in vitro*. J. Agric. Sci.

[15] Conway E.J (1957). Micro Diffusion Analysis and Volumetric Error.

[16] Barnett A.J.G, Reid R.L (1957). Studies on the production of volatile fatty acids from grass and rumen liquor in an artificial rumen 1. Volatile fatty acids production from fresh grasses. J. Agric. Sci.

[17] Kamra D.N, Sawal R.K, Pathak N.N, Kewalramani N, Agarwal N (1991). Diurnal variation in ciliate protozoa in the rumen of blackbuck (*Antilope cervicapra*) fed green forage. Lett. Appl. Microbiol.

[18] IBM SPSS (2011). Statistics for Windows, Version 20.0.

[19] Makkar H.P.S, Blümmel M, Becker K (1995). Formation of complexes between polyvinyl pyrrolidones or polyethylene glycols and tannins, and their implication in gas production and true digestibility in *in vitro* techniques. Br. J. Nutr.

[20] Silanikove N, Perevolotsky A, Provenza F.D (2001). Use of tannin-binding chemicals to assay for tannins and their negative post-ingestive effects in ruminants. Anim. Feed Sci. Technol.

[21] Besharati M, Taghizadeh A (2011). Effect of tannin-binding agents (polyethylene glycol and polyvinylpyrrolidone) supplementation on *in vitro* gas production kinetics of some grape yield byproducts 2011. ISRN Vet Sci.

[22] Haslam E (1989). Plant polyphenols:Vegetable tannins revisited. Chemistry and Pharmacology of Natural Products.

[23] Tang H.R, Covington A.D, Hancock R.A (2003). Structure–activity relationships in the hydrophobic interactions of polyphenols with cellulose and collagen. Biopolymers.

[24] Hatew B, Stringano E, Mueller-Harvey I, Hendriks W.H, Carbonero C.H, Smith L.M.J, Pellikaan W.F (2016). Impact of variation in structure of condensed tannins from sainfoin (*Onobrychis viciifolia*) on *in vitro* ruminal methane production and fermentation characteristics. J. Anim. Physiol. Anim. Nutr.

[25] Gemeda B.S, Hassen A (2015). Effect of tannin and species variation on *in vitro* digestibility, gas, and methane production of tropical browse plants. Asian Australas. J. Anim. Sci.

[26] Huyen N.T, Fryganas C, Uittenbogaard G, Mueller-Harvey I, Verstegen M.W.A, Hendriks W.H, Pellikaan W.F (2016). Structural features of condensed tannins affect *in vitro* ruminal methane production and fermentation characteristics. J. Agric. Sci.

[27] Barahona R, Lascano C.E, Narvaez N, Owen E, Morris P, Theodorou M.K (2003). *In vitro* degradability of mature and immature leaves of tropical forage legumes differing in condensed tannin and non-starch polysaccharide content and composition. J Sci. Food Agric.

[28] Nsahlai I.V, Siaw D.E.K.A, Osuji P.O (1994). The relationships between gas production and chemical composition of 23 browses of the genus Sesbania. J. Sci. Food Agric.

[29] Karabulut A, Canbolat O, Ozkan C.O, Kamalak A (2007). Determination of nutritive value of citrus tree leaves for sheep using *in vitro* gas production technique. *Asian Australas*. J. Anim. Sci.

[30] Ndlovu L.R, Nherera F.V (1997). Chemical composition and relationship to *in vitro* gas production of Zimbabwean browsable indigenous tree species. Anim. Feed. Sci. Technol.

[31] Patra A.K, Saxena J (2011). Exploitation of dietary tannins to improve rumen metabolism and ruminant nutrition. J. Sci. Food. Agric.

[32] Wen L, Wu D, Jiang Y, Prasad K.N, Lin S, Jiang G, He J, Zhao M, Luo W, Yang B (2014). Identification of flavonoids in litchi (*Litchi chinensis* Sonn.) leaf and evaluation of anticancer activities. J. Funct. Foods.

[33] Shukla R.K, Painuly D, Porval A, Shukla A (2014). Proximate analysis, nutritional value, phytochemical evaluation, and biological activity of *Litchi chinensis* Sonn. leaves. J Herbs Spices Med. Plants.

[34] Patra A.K, Saxena J (2010). A new perspective on the use of plant secondary metabolites to inhibit methanogenesis in the rumen. Photochemistry.

[35] dos Santos E.T, Pereira M.L.A, da Silva C.F.P, Souza-Neta L.C, Geris R, Martins D, Santana A.E.G, Barbosa L.C.A, Silva H.G.O, Freitas G.C, Figueiredo M.P (2013). Antibacterial activity of the alkaloid-enriched extract from *Prosopis juliflora* pods and its influence on *in vitro* ruminal digestion. Int. J. Mol. Sci.

[36] Hariadi B.T, Santoso B (2010). Evaluation of tropical plants containing tannin on *in vitro* methanogenesis and fermentation parameters using rumen fluid. J. Sci. Food Agric.

[37] Jayanegara A, Goel G, Makkar H.P.S, Becker K (2010). Reduction in methane emissions from ruminants by plant secondary metabolites:Effects of polyphenols and saponins. Sustainable Improvement of Animal Production and Health.

[38] Yang W.Z, Beauchemin K.A, Rode L.M (2001). Effects of grain processing, forage to concentrate ratio, and forage particle size on rumen pH and digestion by dairy cows. J. Dairy Sci.

[39] Krause K.M, Combs D.K, Beauchemin K.A (2002). Effects of forage particle size and grain fermentability in midlactation cows. II. Ruminal pH and chewing activity. J. Dairy Sci.

[40] Dschaak C.M, Williams C.M, Holt M.S, Eun J.S, Young A.J, Min B.R (2011). Effects of supplementing condensed tannin extract on intake, digestion, ruminal fermentation, and milk production of lactating dairy cows. J. Dairy Sci.

[41] Getachew G, Robinson P.H, De Peters E.J, Taylor S.J (2004). Relationships between chemical composition, dry matter degradation and *in vitro* gas production of several ruminant feeds. Anim. Feed Sci. Technol.

[42] Beuvink J.M.W, Spoelstra S.F (1992). Interactions between substrate, fermentation end products, buffering systems and gas production upon fermentation of different carbohydrates by mixed rumen microorganisms *in vitro*. Appl. Microbiol. Biotechnol.

[43] Moss A.R, Jouany J.P, Newbold J (2000). Methane production by ruminants:Its contribution to global warming. Ann. Zootech.

[44] Santra A, Karim S.A (2002). Influence of ciliate protozoa on biochemical changes and hydrolytic enzyme profile in the rumen ecosystem. J. Appl. Microbiol.

[45] Makkar H.P.S, Francis G, Becker K (2007). Bioactivity of phytochemicals in some lesser-known plants and their effects and potential applications in livestock and aquaculture production systems. Animal.

[46] Bhatta R, Uyeno Y, Tajima K, Takenaka A, Yabumoto Y, Nonaka I, Enishi O, Kurihara M (2009). Difference in the nature of tannins on *in vitro* ruminal methane and volatile fatty acid production and on methanogenic archaea and protozoal populations. J. Dairy Sci.

[47] Newbold C.J, de la Fuente G, Belanche A, Ramos-Morales E, McEwan N.R (2015). The role of ciliate protozoa in the rumen. Front. Microbiol.

[48] Deaville E.R, Green R.J, Mueller-Harvey I, Willoughby I, Frazier R.A (2007). Hydrolyzable tannin structures influence relative globular and random coil protein binding strengths. J. Agric. Food Chem.

[49] González S, Pabon M.L, Carulla J (2005). Effects of tannins on *in vitro* ammonia release and dry matter degradation of soybean meal. Arch. Latinoam Prod. Anim.

[50] Bhatta R, Mani S, Baruah L, Sampath K.T (2012). Phenolic composition, fermentation profile, protozoa population and methane production from sheanut (*Butryospermum parkii*) byproducts *in vitro*. Asian Australas. J. Anim. Sci.

[51] Mamat S.S, Kamarolzaman M.F.F, Yahya F, Mahmood N.D, Shahril M.S, Jakius K.F, Mohtarrudin N, Ching S.M, Susanti D, Taher M, Zakaria Z.A (2013). Methanol extract of *Melastoma malabathricum* leaves exerted antioxidant and liver protective activity in rats. BMC Complement. Altern. Med.

[52] Oskoueian E, Abdullah N, Oskoueian A (2013). Effects of flavonoids on rumen fermentation activity, methane production, and microbial population. BioMed. Res. Int 2013.

[53] Kim E.T, Le Luo Guan S.J.L, Lee S.M, Lee S.S, Lee I.D, Lee S.K, Lee S.S (2015). Effects of flavonoid-rich plant extracts on *in vitro* ruminal methanogenesis, microbial populations and fermentation characteristics *Asian Australas*. J. Anim. Sci.

[54] Goel G, Makkar H.P.S, Becker K (2008). Changes in microbial community structure, methanogenesis and rumen fermentation in response to saponin-rich fractions from different plant materials. J. Appl. Microbiol.

[55] Goel G, Makkar H.P (2012). Methane mitigation from ruminants using tannins and saponins. Trop. Anim. Health Prod.

